# Feature-driven classification reveals potential comorbid subtypes within childhood apraxia of speech

**DOI:** 10.1186/s12887-020-02421-1

**Published:** 2020-11-13

**Authors:** Catherine M. Stein, Penelope Benchek, Gabrielle Miller, Noémi B. Hall, Dhanya Menon, Lisa Freebairn, Jessica Tag, Jennell Vick, H. Gerry Taylor, Barbara A. Lewis, Sudha K. Iyengar

**Affiliations:** 1 Department of Population & Quantitative Health Sciences, Case Western Reserve University, 2103 Cornell Rd, Wolstein Research Building Room 1316, Cleveland, OH 44106 USA; 2 Department of Psychological Sciences, Case Western Reserve University, Cleveland, OH USA; 3 Division of Public Health, Michigan State University, Lansing, MI USA; 4 Cleveland Hearing and Speech Center, Cleveland, OH USA; 5 Department of Pediatrics, Case Western Reserve University, and Rainbow Babies & Children’s Hospital, University Hospital Case Medical Center, Cleveland, OH USA; 6 Nationwide Children’s Hospital Research Institute and Department of Pediatrics, The Ohio State University, Columbus, OH USA

**Keywords:** Speech disorder, Language impairment, Clinical subtypes, Speech severity, Speech-sound disorder, Communication endophenotypes, Developmental comorbidities

## Abstract

**Background:**

Childhood apraxia of speech (CAS) is a neurodevelopmental disorder with heterogeneous communication and other comorbid manifestations. While previous studies have characterized speech deficits associated with CAS, few studies have examined variability in reading and language and/or other developmental comorbidities. We sought to identify comorbid subgroups within CAS that could be clinically relevant as well as genetically distinctive.

**Methods:**

In a group of 31 children with CAS and 8 controls, we performed hierarchical cluster analysis utilizing measures of articulation, vocabulary, and reading. We also conducted a chart review of the children with CAS to examine other clinical characteristics in these children and their association with subgroup membership.

**Results:**

We identified 3 comorbid subgroups within CAS of varying severity. The high severity subgroup was characterized by poor reading and vocabulary, and the moderate severity subgroup by poor reading and non-word repetition but average vocabulary, compared to the mild severity subgroup. Subgroups were indistinguishable with respect to speech sound production, the hallmark of CAS, all demonstrating poor articulation. Children in the most severe subgroup were more likely to have early problems feeding (*p* = 0.036).

**Conclusions:**

Children with CAS may potentially be classified into comorbidity groups based on performance on vocabulary and reading measures, providing additional insight into the heterogeneity within CAS with implications for educational interventions.

**Supplementary Information:**

The online version contains supplementary material available at 10.1186/s12887-020-02421-1.

## Background

Childhood Apraxia of Speech (CAS) is a speech sound disorder (SSD) with a range of severity, resulting in unintelligible speech that often persists into elementary school age and impacts language and literacy skills. Children with CAS demonstrate heterogeneity in their symptoms and diagnostic criteria for the disorder have been highly controversial. The American Speech-Language-Hearing Association (ASHA) has defined CAS as a “…speech disorder in which the precision and consistency of movements underlying speech are impaired in the absence of neuromuscular deficits (e.g., abnormal reflexes, abnormal tone)” [1]. Children with CAS have difficulty selecting and executing motor programs for speech, resulting in inconsistent speech production, difficulty sequencing syllables, and abnormal prosody [1], often accompanied by language and literacy difficulties [1–6] . CAS is a disorder of the central nervous system [2, 7]. The prevalence estimates in the United States range between 0.01–0.02% [8], putting it in the categories of genetic rare disorders, and is reportedly found in 3.4–4.3% of children referred for speech sound disorders [9].

There is controversy in the field whether or not CAS should be classified as a “syndrome”, i.e., a symptom complex, or solely a motor-speech disorder. Velleman [10] states “It is important to note, however, that CAS is a syndrome (i.e., a symptom complex), not a unitary disorder. That is, not all children will demonstrate the same symptoms and symptoms will change over time in a given individual. Thus, a checklist approach to diagnosis is not possible; rather, a pattern of symptoms is key to identification” (p.59). Gillon and Moriarty [3] refer to CAS as a “symptom cluster of speech, motor, and/or language characteristics” and note that there is no single characteristic that can be used in isolation to diagnose it. The concept of CAS as a syndrome is further discussed by Nijland et al. [11]. While CAS may co-occur with known neurodevelopmental disorders and/or dysarthria [12], it may also be caused by genetic mutations, many of which are still uncharacterized [13, 14], or it may be idiopathic. A genetic study of two multigenerational families with histories of SSD associated with CAS revealed findings suggesting a spectrum of CAS phenotypes [13]. Another genetic study of 10 unrelated participants with CAS observed significant genetic heterogeneity and a high degree of phenotypic diversity [14].

As stated by Stackhouse and Wells [15], the motoric deficit seen in CAS may have “flow-on” effects for a child’s language and literacy development. Inconsistent and inaccurate speech output in CAS may provide inadequate input to the child’s developing linguistic system and may thus also affect auditory processing and subsequent vocabulary knowledge. This cascading effect of motor-speech difficulties on a child’s developing language and literacy development may impact linguistic abilities and the ensuing reading and spelling skills for school-age children with CAS [1, 6, 16, 17]. Thus, an effect of motor-speech impairment on language and literacy is that poor speech output interferes with the accurate encoding of complex words, which affects the child’s developing lexicon and linguistic system [15]. Previous studies have demonstrated language and reading disabilities of varying prevalence and severity among children with CAS [3–6]. This illustrates how articulation, language, and reading are inter-dependent.

In addition to characteristic difficulties in speech production and associated reading and language difficulties, CAS may also present with other neurological signs, including early difficulties with feeding, sensory processing issues, a paucity of vocal play, babbling, and imitation in infancy, gross or fine motor in-coordination, body dyspraxia, dysarthria, and other “soft” neurological signs in addition to slow progress in therapy and limited repertoire of sounds [10, 11, 18–21]. These clinical characteristics present differently at various stages of child development and are highly variable across children with CAS. While many papers have characterized the speech deficits associated with CAS, few studies have explicitly examined these other clinical characteristics. As illustrated above, there is much to be learned about the phenotypic diversity of CAS, which could potentially reveal clues about its biologic underpinnings.

Because of the potential heterogeneity within CAS manifested in difficulties in written language [3, 11, 22–24], it is of interest to see if there are subgroups within CAS; this could have implications for treatment of other communication domains. There is no widely agreed-upon metric to characterize CAS severity. Comorbid disorders that occur with CAS have not been utilized for CAS as a means of forming severity subgroups. Our objective was to use speech, language, and reading assessments to identify comorbid subgroups within CAS of varying severity and associated clinical characteristics, based on the aforementioned literature suggesting that these are variable within children with CAS. Specifically, we exploited the full range of phenotypic variation to identify more homogeneous subgroups of children, an approach that was advocated for autism (another neurodevelopmental disorder) several years ago for genetic mapping [25]. Subgroup classification of CAS and other communication disorders has been a long-standing interest for communication disorders professionals as it may potentially lead to differential diagnosis and treatment [3, 26]. In addition, recognition of clinical characteristics associated with varying severity levels within CAS could help identify children in need of early or more intense remediation, thus improving later academic outcomes. First, using endophenotypes of communication disorder severity, specifically articulation, vocabulary, phonological memory and reading, we used an unsupervised multivariate clustering method to mine the data for potential comorbid subgroups. Then, we conducted a chart review of other clinical symptoms associated with CAS to examine if these symptoms were associated with comorbid subtypes of CAS as defined by the degree of impairment in language and reading ability. This analysis presents an unusually large cohort of children with CAS. Together, these data suggest that there may be comorbid subgroups within CAS that can be defined by language and reading ability as well as the presence of specific clinical symptoms.

## Methods

### Participants

In this study, we examined 31 individuals diagnosed with childhood apraxia of speech (CAS) as part of the Cleveland Family Speech and Reading Study [4, 27–31] (Supplemental Table 1). Children with CAS were identified from caseloads of speech-language pathologists in the Greater Cleveland area and referred to the study between 1991 and 2003. All participants met inclusion criteria based on information provided by a parent in an interview or via questionnaire including: normal hearing acuity; fewer than six episodes of otitis media prior to age 6; monolingual English speaker; absence of a history of neurological disorders other than CAS, such as cerebral palsy or autism spectrum disorder; and a diagnosis of a SSD or suspected CAS by a local speech-language pathologist or neurologist. The diagnosis of CAS was confirmed based on direct testing of motor speech and articulation by an experienced licensed speech-language pathologist upon enrollment into the study. Complete details on the diagnosis of CAS are provided in the Supplemental Methods. All children with CAS in this analysis were unrelated. For the cluster analysis described below, 8 additional individuals were randomly selected from those study participants who were unaffected for speech sound disorder and/or language impairment based on parent report and our independent assessment (Supplemental Table 2). Each child in the study was given a battery of tests to assess articulation, vocabulary, phonological memory, and reading as described below. As data were obtained as part of a larger longitudinal study, test scores were based on the initial administration of each measure. If a child could not complete a test due to age, we utilized an assessment from a later age (next visit) for that measure, and age-adjusted accordingly (see Statistical methods, described below). Socioeconomic status was determined at the initial assessment based on parent education levels and occupations using the Hollingshead Four Factor Index of Social Class [32]. In addition, parent interviews were conducted to collect information about the child’s medical and developmental history. Presence of ADHD was determined by parent report based on the diagnosis by a psychologist or neurologist. Reading disability (RD) was determined if the child was receiving reading services in the schools, and language impairment (LI) was determined by the diagnosis of a speech-language pathologist. This study was approved by the Institutional Review Board of Case Medical Center and University Hospitals and all parents provided informed consent and children provided written informed assent.

### Communication and cognitive measures

We examined articulation using the *Goldman-Fristoe Test of Articulation (GFTA)-Sounds in Words subtest* [33, 34] and diadochokinetic rates using the *Robbins and Klee Oral Speech Motor Control Protocol* [35] or *Fletcher Time-by-Count Test of Diadochokinetic Syllable Rate* [36] *.* The Robbins and Klee was reverse scored prior to being merged with scores from the Fletcher Time-by-Count Test. For the merged variable, referred to as the Diadochokinetic Syllable Rate or DDK, higher scores reflect better performance. We excluded the DDK measure from the cluster analysis, because the scores were uniformly low among participants with CAS with little variability. Inclusion of such a variable within the multivariate analysis would have concealed any difference among children with CAS because they all had poor scores particularly in contrast with the normal children.

Expressive vocabulary was assessed with the *Expressive One Word Picture Vocabulary Test-Revised* (*EOWPVT* [37]*)* and receptive vocabulary with the *Peabody Picture Vocabulary Test- Third Edition (PPVT* [38]*),* and phonological memory with the *Nonsense word repetition task (NWR* [39]*)*. Reading was assessed using the *Woodcock Reading Mastery Test-Revised, Word Attack subtest (WRMT-AT* [40]) and *Word Identification Subtest (WRMT-ID* [40]*)*.

*Performance IQ (PIQ)* was assessed by the *Wechsler Preschool and Primary Scale of Intelligence- Revised (WPPSI-R)* or the *Wechsler Intelligence Scale for Children- III (WISC-III; Wechsler, 1991)* [41, 42]. These tests measure cognitive skills such as problem solving, spatial perception, working memory, and visual-motor co-ordination. Subtest scores were combined to form a PIQ score.

Children were not penalized for speech sound errors on the expressive vocabulary measure (EOWPVT) or the spoken reading measures (Word Attack or Word Identification). Rather, if they identified the picture correctly on the EOWPVT they received credit regardless of speech errors. Similarly, if they read the word aloud correctly they also received credit regardless of speech errors. Graphical illustration of NWR scores illustrates that children did not fail this task because of articulation issues associated with CAS (Supplemental Fig. 1). (See Supplemental Methods for additional information on these measures.)

### Clinical and family characteristics

Clinical characteristics of children with CAS, including reports of motor in-coordination, sensory processing issues, early feeding difficulties, little vocal play, babbling or imitation, limited repertoire of sounds, body dyspraxia, and dysarthria (see Table 1) were obtained in reviewing children’s medical and developmental history as part of parent interviews; these parental reports were not confirmed clinically. Family histories of SSD, language impairment (LI), and reading disorder (RD) were also obtained via parent interview.

**Table 1:** Demographic and clinical characteristics of children with CAS

Age at enrollment (mean (SD))^a^	5.9 (2.5)
Proportion female	19%
Proportion with language impairment (LI)	94%
Proportion with reading disability (RD)	58%
Proportion with ADHD	48%
Socioeconomic status according to Hollingshead four factor index of social class [32]	
1 (lowest)	0
2	3%
3	42%
4	32%
5 (highest)	23%

^a^Note that the age at enrollment might not necessarily correspond with the age at first assessment. Some children were diagnosed with CAS at a young age and were too young to do the testing

### Statistical methods

Age-adjusted standard scores for EOWPVT, GFTA, PPVT, WRMT-AT, WRMT-ID, and DDK tests, as provided with the tests, were transformed to z-scores (mean = 0, SD = 1). Because there are no normative data for the NWR, Z-scores for the NWR were created by regressing raw scores on age in the subsample of unaffected siblings of probands from the larger Cleveland Family Study cohort. The resulting regression equations were used to derive age-adjusted NWR scores, as in our previous work [29, 43, 44]. Because examination of clinical and family characteristics associated with the CAS severity subgroups was exploratory in nature, a nominal *p*-value less than 0.05 was regarded as significant.

All analyses were conducted using R software. For the cluster analysis we used hierarchical clustering (hclust). A small sample (*N* = 8) of controls was selected because the goal was to differentiate among the children with CAS. Many clustering methods tend towards equal cluster sizing, so a larger sample of controls would have overwhelmed the analysis, leading to no differentiation among subgroups within the children with CAS. A multivariate matrix of 6 communication traits (EOWPVT, PPVT, NWR, GFTA, WRMT-AT, WRMT-ID) was constructed for all the subjects with CAS plus 8 controls, so that individuals with similar scores clustered together. Assignment of separate clusters was based on dissimilarity across the six test scores. Dissimilarity was determined using Euclidean distance and clustering was done using complete linkage. The number of clusters was determined by visualizing the dendrogram (cluster tree) and cluster size was not predetermined. After identification of clusters, the distribution of the various scores for the individuals within each group were compared using the Kruskal-Wallis test for overall difference in distributions across all groups and the Mann-Whitney test for pairwise differences, and the distribution of the presence of clinical and family characteristics with subgroup membership was examined using Fisher’s exact test. As a follow-up analysis, we compared PIQ scores across these clusters using pairwise Welch’s t-tests and ADHD prevalence across clusters using Fisher’s exact test. Lastly, to evaluate the stability of these severity groups with developmental trajectories on these tests, we compared the distribution of test scores across these groups with values taken at the last available assessment (when the children were teenage in most cases).

## Results

We examined 31 individuals with CAS, of whom 19% were female, 94% had comorbid language impairment and 58% had reading disability (Table 1 and Supplemental Table 1). The average age at first assessment was 5.9 years (standard deviation 2.5). Males and females did not differ significantly at the α = 0.05 level on test scores or clinical/family characteristics (Supplemental Table 3).

**Fig. 1:**
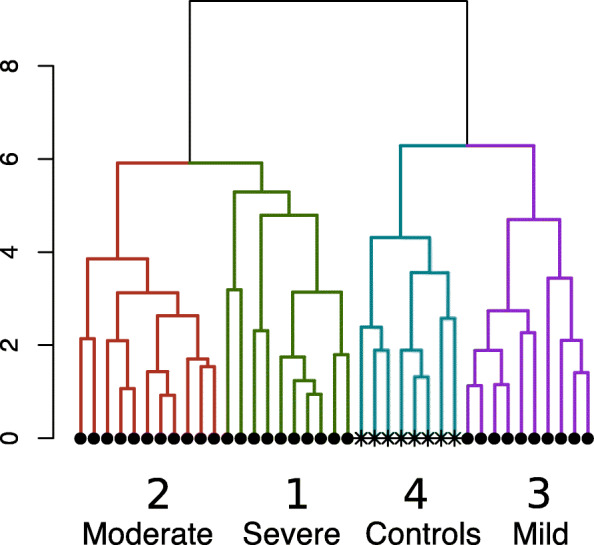
Cluster analysis dendogram illustrating clustering of subjects. Circles indicate apraxic children, asterisks (*) indicate typical children. Cluster assignment is indicated using color and the numbering scheme below. The y-axis shows the estimated Euclidean distance that was used for the clustering algorithm

**Fig. 2:**
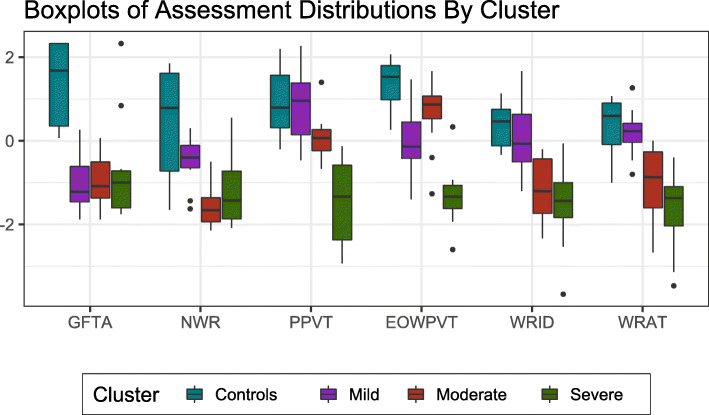
Distribution of trait scores by cluster membership. NWR = nonsense word repetition, GFTA = Goldman-Fristoe test of Articulation, PPVT = Peabody Picture Vocabulary Test, EOWPVT = Expressive one word picture vocabulary text, WRMT-ID – Weschsler Reading Mastery Test, Word identification subtest, and WRMT-AT = Weschsler Reading Mastery Test, Word attack subtest

Next, we examined how children with CAS clustered based on their scores on articulation, language, and reading endophenotypes. Descriptive statistics for children in the “control” group are shown in Supplemental Table 2. While DDK was excluded from the multivariate cluster analysis, we provide descriptive statistics by cluster for the reader’s interest. Analysis revealed 4 distinct clusters (Fig. 1) that were well separated. Three of these clusters consisted exclusively of individuals with CAS, while the 8 controls formed their own cluster. The mean score for each variable by cluster membership is shown in Fig. 2, illustrating meaningful phenotypic differences between the clusters. The three comorbid subgroups within CAS were divided into mild, moderate, and high severity, based on performance on these measures. The three clusters of children with CAS were indistinguishable based on the articulation task (GFTA), though these three clusters had significantly lower scores on this measure than the control group (*p* < ≤0.004). The lack of differentiation on GFTA is consistent with the literature [45, 46]. Similarly, analyses also failed to reveal significant differences among the comorbid CAS subgroups on DDK, and all of these subgroups differed significantly from the control group (*p* ≤ 0.003) (Table 2). The mild severity comorbid CAS subtype group (#3) performed better than the moderate severity comorbid CAS subtype group (#2) on nonsense words (NWR) (*p* < 0.001) and on the two reading tasks, WRMT-ID (*p* = 0.004) and WRMT-AT (*p* = 0.001). Interestingly, the mild severity comorbid subtype group did not differ significantly from the controls on NWR, Peabody Picture Vocabulary Test (PPVT), or either reading measure (WRMT-ID and WRMT-AT) (*p* ≥ 0.20). The high and moderate severity comorbid CAS subtype groups (#1 and #2) had similar mean scores on GFTA, NWR, WRMT-ID and WRMT-AT but the high severity comorbid CAS subtype group differed significantly from the mild and moderate severity CAS subgroups (#2 and #3) on the vocabulary tasks, EOWPVT and PPVT (all *p* < 0.003). To examine whether cluster assignment was determined by the age of the child during the assessment, we examined the mean ages within cluster for the reading (WRMT-AT and WRMT-ID) and vocabulary (EOWPVT and PPVT) assessments, which were the two domains that differentiated the clusters (Supplemental Table 5). There was no significant difference across clusters with respect to age at reading assessment, and among the CAS clusters, there was no significant difference with respect to age at vocabulary assessment.

**Table 2:** Descriptive statistics for articulation, vocabulary, and reading tasks by cluster

Variable	Cluster 1 (High severity) (*n* = 10)	Cluster 2 (Moderate severity) (*n* = 11)	Cluster 3 (Low severity) (*n =* 10)	Cluster 4 (Controls) (*n* = 8)	Kruskal-Wallis *P-*value^1^	Mann-Whitney exact *P-*value^2^	Mann-Whitney exact *P-*value^3^	Mann-Whitney exact *P-*value^4^	Mann-Whitney exact *P-*value^5^	Mann-Whitney exact *P-*value^6^	Mann-Whitney exact *P-*value^7^
					*All clusters*	*Cluster 1* vs *2*	*Cluster 2* vs *3*	*Cluster 3* vs *4*	*Cluster 1* vs *3*	*Cluster 2* vs *4*	*Cluster 1* vs *4*
EOWPVT	−1.32 (0.76) [−1.33]	0.65 (0.83) [0.87]	0.05 (0.85) [−0.13]	1.37 (0.60) [1.53]	< 0.001	< 0.001	0.12	0.003	0.003	0.03	< 0.001
GFTA	−0.70 (1.31) [−1.00]	− 0.96 (0.69) [− 1.08]	−1.00 (0.76) [− 1.21]	1.38 (1.05) [1.68]	0.002	0.96	0.93	< 0.001	0.81	< 0.001	0.004
NWR	−1.17 (0.89) [− 1.42]	−1.58 (0.50) [− 1.66]	− 0.48 (0.63) [− 0.39]	0.39 (1.40) [0.79]	0.002	0.31	< 0.001	0.20	0.09	0.002	0.02
PPVT	−1.45 (1.02) [− 1.33]	0.08 (0.56) [0.07]	0.79 (0.91) [0.97]	0.94 (0.85) [0.80]	< 0.001	< 0.001	0.07	0.70	< 0.001	0.01	< 0.001
WRMT-AT	−1.66 (0.99) [− 1.37]	−1.07 (0.92) [−0.87]	0.19 (0.58) [0.23]	0.37 (0.71) [0.60]	< 0.001	0.23	0.001	0.53	< 0.001	0.003	< 0.001
WRMT-ID	−1.55 (1.01) [− 1.43]	−1.16 (0.79) [− 1.20]	0.05 (0.86) [−0.07]	0.37 (0.54) [0.47]	< 0.001	0.46	0.004	0.37	< 0.001	< 0.001	< 0.001
DDK	−8.60 (7.08) [−7.35]	−7.81 (7.89) [−5.00]	−7.21 (7.48) [−6.84]	−0.07 (0.68) [− 0.15]	0.003	0.81	0.99	< 0.001	0.68	0.002	0.003

Includes both CAS and controls. Values shown as mean (sd) [median]. *P-*values shown are not adjusted for multiple testing. ^1^Comparison of distribution across all four clusters. ^2^Comparison of distribution medians between clusters 1 and 2. ^3^Comparison of distribution between clusters 2 and 3. ^4^Comparison of distribution between clusters 3 and 4. ^5^Comparison of distribution between clusters 1 and 3. ^6^Comparison of distribution between clusters 2 and 4. ^7^Comparison of distribution between clusters 1 and 4

To examine the stability of these severity groups with respect to changes that may occur with age and resolution of CAS, we repeated the descriptive analysis described above, only this time utilizing the last available assessment for each measure (Supplemental Table 4). In most cases, these observations were taken when the children were now teenagers. We found that the pattern of differences seen between the severe, moderate, and mild groups was maintained when examining these later time points. This analysis suggests that classification of severity of CAS as defined by these measures is robust to age. We also evaluated the impact of including the small sample of children unaffected for speech and language as a control group in the cluster analysis. We repeated the analysis without the control group, and the cluster membership of children with CAS remained the same (Supplemental Fig. 2).

Because vocabulary is highly correlated with IQ (correlation between PIQ and PPVT = 0.64 in this sample), we followed-up our examination of these comorbid subgroups by comparing PIQ across groups. We found that the mild subgroup was significantly different than the most severe group (*p* = 0.03), and the control group differed significantly from the severe (*p* = 0.01) and moderate (*p =* 0.01) subgroups (Supplemental Fig. 3). These findings suggest that the mild comorbid subgroup may have a higher PIQ than the more severe subgroups. Given that poor attention affects memory tasks on the PIQ, we examined the prevalence of ADHD across these comorbidity subgroups. We found that ADHD was significantly more common the severe comorbidity subgroup (80%) compared to the moderate severity (45%) and mild severity (20%) and control group (12.5%) (overall *p* = 0.013). (Supplemental Table 6).

When comparing clinical characteristics of the three comorbid CAS subtype groups, the high severity subgroup (#1) had a significantly higher prevalence of early problems with feeding than members of the other two groups (50% (#1) versus 27% (#2) and 0% (#3), *p* = 0.036) (Table 3). Fine motor in-coordination was also more common in the high severity comorbid subtype group compared to the mild and moderate severity comorbid subtype groups (50% versus 18 and 20% respectively), although this difference was not statistically significant. While the cluster sizes were small, there was also a significant difference in the distribution of sex across the 3 groups; the moderate severity subgroup (#2) had no females, while the other two subgroups included females (*p* = 0.02) (Table 4). There were no differences with respect to SES or family history of SSD, LI, and/or RD (Table 4). Because only one non-Caucasian child was present in the sample, we did not compare groups with respect to race.

**Table 3:** Association Between Clinical Indicators in CAS Individuals and Cluster Assignment

Clinical Variable	Cluster 1 (High severity) (*n* = 10)	Cluster 2 (Moderate severity) (*n* = 11)	Cluster 3 (Mild severity) (*n* = 10)	Fisher exact *P-*value^1^
Problems with Feeding Eating	0.50	0.27	0	**0.036**
Little vocal play or babbling	0	0.18	0.20	0.511
Family history of communication disorders	1	0.91	1	0.999
Delayed language onset	0.50	0.73	0.80	0.398
Gross motor incoordination	0.30	0.27	0.30	0.999
Fine motor incoordination	0.50	0.18	0.20	0.298
Body dyspraxia body awareness in space	0.10	0.09	0	0.999
“Soft” neurological signs	0.10	0.27	0	0.289
Sensory processing issues	0.20	0	0.20	0.353
Dysarthria	0.10	0.27	0.20	0.849
Limited repertoire of sounds	0.20	0.64	0.50	0.128

Values shown are proportion with the trait. “n” is the number of CAS individuals per cluster. *p*-values shown are not adjusted for multiple testing. ^1^Test of association is across CAS clusters 1,2 and 3 only

**Table 4:** Association Between Descriptive Variables and Cluster Assignment in CAS and Controls

Descriptive Variable/Parental Trait		Cluster 1 (High severity) (*n =* 10)	Cluster 2 (Moderate severity) (*n =* 11)	Cluster 3 (Mild severity) (*n =* 10)	Cluster 4 (Controls) (*n =* 8)	Fisher exact *P*-value^1^
SEX (Female)		0.30	0	0.30	0.63	0.02
SES	Stratum					0.45
	1	0	0	0	0	
	2	0.10	0	0	0.14	
	3	0.30	0.55	0.40	0.14	
	4	0.40	0.27	0.30	0.71	
	5	0.20	0.18	0.30	0	
Race (Caucasian)		0.90	1	1	1	0.72
Language Disorder in Either Parent		0.10	0	0.20	0.13	0.50
Reading Disorder in Either Parent		0.40	0.27	0.20	0	0.26
Speech Disorder in Either Parent		0.60	0.55	0.50	0.50	0.99

Values shown are proportion with the trait. “n” is the number of CAS and control individuals per cluster. *p*-values shown are not adjusted for multiple testing. ^1^Test of association is across all 4 clusters

## Discussion

The goal of this study was to examine whether comorbid subtypes within CAS could be classified based on variability in reading, vocabulary, and articulation. Identification of more homogenous subgroups has potential utility in identification of causal genetic variants [25] and in understanding potential cognitive and educational outcomes for children with CAS. Our analysis revealed 3 phenotypic subgroups with deficits of varying severity in language and reading skills. The high severity comorbid CAS subtype group performed the worst with respect to vocabulary compared to the other two subgroups, and the moderate severity comorbid CAS subtype group had poorer outcomes than the mild severity subgroup on measures of reading and non-word repetition. Consistent with our previous findings [6], the results suggest that comorbid subtype severity within CAS is manifested in deficits in language and phonological processing skills associated with reading disability. We found that these subgroups were robust to age, as there were consistent differences between groups when examining assessments taken at later ages. In addition, the high severity comorbid subtype group had a significantly higher prevalence of early feeding issues and ADHD, and potentially more fine motor problems, although this difference did not achieve statistical significance. Both results suggest the possibility of more pervasive motor deficits in the high severity comorbid subtype group of children. Feeding difficulties have long been reported in children with CAS [20, 47, 48] and may indicate a global motor apraxia. This understanding of a constellation of deficits in written and spoken language is important for clinical care and educational planning.

Our findings further suggest that CAS is a heterogeneous disorder [3, 10, 23]. Future studies of CAS may reveal a wide range of phenotypic manifestations associate with mutations in the same gene, as has been found for other developmental disabilities [49, 50]. Examination of clinical characteristics revealed that all children with CAS in our sample had a family history of communication disorders, supporting a role for genetic factors in the etiology of CAS. The findings also revealed that, while less-often endorsed than a family history of communication disorders, most children with CAS had delayed onset of language. Consistent with previous findings, results additionally document associations of vocabulary knowledge as a marker of CAS comorbid severity [26]. Vocabulary is one of many components that are core to speech sound acquisition. Finally, while CAS is more common in males, individual endophenotypes and clinical characteristics did not differ significantly in males compared to females. Sex differences are much more pronounced among children with other types of speech sound disorder [51], so this is a notable dissimilarity.

The lack of differences between the comorbid CAS subtype groups in articulation and oral-motor skills suggests that assessment of other skills is needed to predict outcomes for children with CAS, which is consistent with the idea that no single characteristic can be used to differentiate children with CAS [3]. Poor vocabulary scores were a key marker of severity, hence vocabulary knowledge is likely to be useful in early identification of the children at highest risk for more severe language and reading disabilities [52]. Children in the high comorbid severity CAS subgroup also had a higher prevalence of early feeding difficulties than the other two subgroups, thus this clinical feature of the disorder may also be useful in predicting further language and reading difficulties and poorer outcomes. The results underscore the importance of early monitoring of language and phonological skills and suggest that remediation focusing on language and phonological therapy in addition to speech sound production may be useful in avoiding or attenuating later academic problems [5]. Associations of the severity of CAS with vocabulary knowledge is consistent with findings from a study of children at familiar risk for dyslexia by Viholainen et al. that found that subgroups identified via familial risk for dyslexia and presence of early motor developmental issues had poorer vocabulary and inflectional morphology. Based on their findings, the researchers recommended early interventions to promote language development [53].

Nijland et al. [11], who examined a variety of cognitive characteristics in children with and without CAS, conceptualized this disorder as a symptom complex comprised of errors at different levels of speech processes related to multiple underlying deficits, rather than as a unitary disorder. Our work supports their hypothesis by showing there are varying levels of reading and vocabulary deficits among children with CAS, accompanied by other clinical characteristics related to motor function. A second view of CAS has been as a motor-speech disorder with a core linguistic deficit. The core linguistic deficit may impact phonological representations and/or the neurological organization of both language and speech [11, 54–59]. Further, the relationship of LI and RD to the diagnosis of CAS has been controversial. CAS, for instance, has been viewed primarily as a disorder of speech-motor planning; LI and RD are not included in the definition of CAS but are thought to be simply co-occurring [1, 21, 60, 61]. Third, some researchers have gone further to define CAS as a syndrome, i.e. a constellation of difficulties having a single cause of either known or unknown etiology [10, 23, 62]. The CAS syndrome is a complex neurodevelopmental disorder with deficits in motor-speech, cognition, language and literacy [11, 13, 14, 30, 63, 64]. Our findings, demonstrating that reading and language difficulties vary among subgroups within children with CAS, are less concordant with the second conceptualization than the first and third.

CAS being a rare disorder, results in one limitation of the study, the sample size is necessarily small. A second limitation is that test scores, although age adjusted, were obtained at different ages depending on when children were seen for follow-up as part of the larger longitudinal project. Additionally, clinical and family characteristics were based on parent report and thus subject to recall bias, and this information was not available for control participants. A third limitation was that the control group was predominantly female, though this is not surprising, since both CAS and SSD are more common in males, so a randomly-selected control group would more likely be predominantly female. Because of this demographic difference, comparisons between comorbid subtype groups could be biased towards the null hypothesis (i.e. not statistically significant when differences may in fact exist), but with so few females in the affected groups, this analysis is underpowered nonetheless. While comparisons among children with CAS could be underpowered because of the rarity of female cases, they are still of interest to the field.

## Conclusion

In summary, multivariate cluster analysis of scores of children with CAS on tests of vocabulary, nonword repetition, and reading revealed potential comorbid subgroups of varying severity. The subgroups suggest that comorbid subtypes within CAS of differing degrees of severity may be distinguished based on these features. The most severe comorbid CAS subtype group also had higher rates of early difficulties in feeding and fine motor in-coordination than the other subgroups, indicating that these difficulties may also be useful in predicting comorbid CAS severity. The severe group also has a greater prevalence of ADHD. This heterogeneity of CAS implied by identification of these subgroups may be related to differences in neural development and associated with genetic variability [7] The findings support a need to design early interventions tailored to the different profiles of deficits associated with CAS [3].

## Supplementary Information

**Additional file 1.**

**Additional file 2.**

## Data Availability

The datasets used and/or analysed during the current study are available from the corresponding author on reasonable request.

## Abbreviations

ADHD: Attention-deficit Hyperactivity Disorder
ASHA: American Speech-Language Hearing Association
CAS: Childhood apraxia of speech
DDK: Diadochokinetic Syllable Rate
EOWPVT: Expressive One Word Picture Vocabulary Test
GFTA: Goldman-Fristoe Test of Articulation
LI: Language impairment
NWR: Nonword repetition
PIQ: Performance Intelligence Quotient
PPVT: Peabody Picture Vocabulary Test
RD: Reading disability
SSD: Speech sound disorder
WRMT-AT: Woodcock Reading Mastery Test – Word Attack
WRMT-ID: Woodcock Reading Mastery Test – Word Identification
