# Supplementary material for: Feature-driven classification reveals potential comorbid subtypes within childhood apraxia of speech
Source: BMC Pediatr. 2020 Nov 13;20:519. doi: 10.1186/s12887-020-02421-1 (PMC7664029; doi:10.1186/s12887-020-02421-1)
Supplement: Supplementary file 1 — Additional file 1. [file 12887_2020_2421_MOESM1_ESM.docx]

SUPPLEMENTAL METHODS

**Diagnosis of CAS**

A subset of children who were enrolled in the Cleveland Family Speech and Reading Study had a history of CAS diagnosis (and treatment) from experienced speech-language pathologists. Upon entry into the study, this diagnosis was confirmed by the expert opinion of two ASHA certified speech-language pathologist serving on the research team with experience in motor-speech disorders [1, 2]. Often, the speech-language pathologists on the research team found that the prior diagnosis of CAS was too liberal, and these children were reclassified for the research study as not having CAS. The two expert speech-language pathologists conducted their reviews independently, and were always in agreement. The participants were required to exhibit a minimum of four of the following seven features of CAS as reported in the literature either in conversational speech or elicited through direct testing.

1) Greater difficulty with polysyllabic words than with monosyllabic words. Syllabic integrity is affected by omissions of syllables, and consonants, and cluster simplification [1, 3-8].

2) Restricted phonemic repertoire and reduction in syllable shape [9, 10].

3) Atypical phonological errors (e.g., initial consonant deletion, voicing and unvoicing errors, glottal replacement) [3, 8, 11, 12].

4) A combination of 2 and three sound feature errors, prolongations, and repetitions of sounds or syllables, distortions, omissions, and additions in repetitive speech tasks or conversational speech [8].

5) Abnormal prosody, slow rate, and equal stress especially in older children or those who have received speech therapy [8, 13, 14].

6) Metathetic errors [15, 16].

7) Reduced diadochokinetic rates often produced with incorrect syllable sequence [1, 8, 17].

**Measures**

*The Goldman-Fristoe Test of Articulation (GFTA)-Sounds in Words subtest [18]* is a series of pictures with target words that assesses children’s ability to produce 39 sounds of the English language in various locations of the word (initial, medial, final) as well as consonant blends in the initial position [18]. Responses were audio recorded and phonetically transcribed. Over the course of this study, two different versions of this test have been administered; standard scores were created for the purposes of this analysis. This test is appropriate for ages 2-21 years.

The *Robbins and Klee Oral Speech Motor Control Protocol* [19] (DDK-OSMCP) assessed oral motor skills in children 4 to 6 years. Diadochokinetic rates on single, double and multi-syllables were timed and scored. The *Fletcher Time-by-Count Test of Diadochokinetic Syllable Rate [20]* (DDK-FL) was administered to children 7 years or older. This test also assesses rates of syllable repetition on single, double and multi-syllable words. Z-scores were generated, and the scores for single and multi-syllables were combined for analysis. Because the DDK-OSMCP is scored in the opposite direction (low scores indicating better performance), these values were negated prior to merging with the DDK-FL; for this merged score, lower scores imply worse performance. We shall refer to this merged variable as DDK.

*Expressive One Word Picture Vocabulary Test-Revised* (*EOWPVT [21])* assesses expressive vocabulary and requires the examinee to name pictures representing objects, actions, and concepts. *Peabody Picture Vocabulary Test- Third Edition (PPVT-III [22])* tests receptive vocabulary and requires the examinee to point to the image that best matches the stimulus from a group of four*.*  Both of these measures are given to children ages 2 years or older.

*Nonsense word repetition (NWR [23])* requires children to repeat 15 non-words, a task which requires encoding of unfamiliar phonological sequences; deficits in encoding would result in inaccurate word repetition[23] and can discriminate children and adults with resolved SSD from those who never had SSD [24]. While this task is normally given to individuals ages 4 years through adulthood, the children with CAS generally could not perform this task in preschool, so the first available assessment starting at age 7 was used for this analysis.

The *Woodcock Reading Mastery Test-Revised, Word Attack subtest (WRMT-AT [25]*) evaluates phonetic decoding skills by requiring examinees to read a list of 45 non-words; the test includes nonwords such as *ip, din, ceisminadolt, and gnouthe*. The *Woodcock Reading Mastery Test- Revised, Word Identification Subtest (WRMT-ID [25])* assesses single word reading ability by requiring examinees to read a list of 106 real words. This task is given to individuals ages 5 through adulthood.

REFERENCES

1. Murray E, McCabe P, Heard R, Ballard KJ: **Differential diagnosis of children with suspected childhood apraxia of speech**. *J Speech Lang Hear Res* 2015, **58**(1):43-60.

2. Duffy JR: **Motor speech disorders: substrates, differential diagnosis, and management**. In*.*: Mosby; 2013.

3. Lewis BA, Freebairn LA, Hansen AJ, Iyengar SK, Taylor HG: **School-age follow-up of children with childhood apraxia of speech**. *Language, speech, and hearing services in schools* 2004, **35**(2):122-140.

4. Jacks A, Marquardt TP, Davis BL: **Consonant and syllable structure patterns in childhood apraxia of speech: developmental change in three children**. *J Commun Disord* 2006, **39**(6):424-441.

5. Marquardt TP, Sussman HM, Snow T, Jacks A: **The integrity of the syllable in developmental apraxia of speech**. *J Commun Disord* 2002, **35**(1):31-49.

6. Rosenbek JW, R;: **A review of fifty cases of developmental apraxia of speech.** *Lang Speech and Hear Services in Schools* 1972, **3**(1):23-33.

7. Velleman SL: **Childhood apraxia of speech resource guide**. Clifton Park, NY: Thompson Delmar Publishing; 2003.

8. Yoss KA, Darley FL: **Developmental apraxia of speech in children with defective articulation**. *J Speech Hear Res* 1974, **17**(3):399-416.

9. Davis BV, SL: **Differential diagnosis and treatment of developmental apraxia of speech in infants and toddlers**. *The Transdisciplinary Journal* 2000, **10**(3):177-192.

10. Overby M, Caspari SS: **Volubility, consonant, and syllable characteristics in infants and toddlers later diagnosed with childhood apraxia of speech: A pilot study**. *J Commun Disord* 2015, **55**:44-62.

11. Baumann R, Kaempfer S, Chegou NN, Nene NF, Veenstra H, Spallek R, Bolliger CT, Lukey PT, Van Helden PD, Singh M *et al*: **Serodiagnostic markers for the prediction of the outcome of intensive phase tuberculosis therapy**. *Tuberculosis (Edinb )* 2013, **93**(2):239-245.

12. Skinner-Meredith ASA: **Cleft lip and palate**. In: *Comprehensive perspectives on speech sound development and disorders: Pathways from linguistic theory to clinical practice.* edn. Edited by Peter BM, AAN. Hauppauge, NY: Nova Science Publishers; 2013: 387-410.

13. **Childhood Apraxia of Speech [Technical Report]**

14. Shriberg LD, Aram DM, Kwiatkowski J: **Developmental apraxia of speech: II. Toward a diagnostic marker**. *J Speech Lang Hear Res* 1997, **40**(2):286-312.

15. Shriberg LD, Austin D, Lewis BA, McSweeny JL, Wilson DL: **The speech disorders classification system (SDCS): extensions and lifespan reference data**. *J Speech Lang Hear Res* 1997, **40**(4):723-740.

16. Shriberg LD, Campbell TF, Karlsson HB, Brown RL, McSweeny JL, Nadler CJ: **A diagnostic marker for childhood apraxia of speech: the lexical stress ratio**. *Clinical linguistics & phonetics* 2003, **17**(7):549-574.

17. Strand EA, McCauley RJ, Weigand SD, Stoeckel RE, Baas BS: **A motor speech assessment for children with severe speech disorders: reliability and validity evidence**. *J Speech Lang Hear Res* 2013, **56**(2):505-520.

18. Goldman R, Fristoe M: **The Goldman-Fristoe test of articulation**. In*.* Circle Pinesm MN: American Guidance Service; 1986.

19. Robbins J, Klee T: **Clinical assessment of oropharyngeal motor development in young children**. *Journal of Speech and Hearing Research* 1987, **52**:271-277.

20. Fletcher D: **The Fletcher Time-by-count test of diadochokinetic syllable rate**. In*.* Tigard, OR: C.C. Publications, Inc.; 1977.

21. Gardner M: **Expressive One Word Picture Vocabulary Test-Revised**. In*.* Novato, CA: Academic Therapy Publications; 1990.

22. Dunn L, Dunn L: **Peabody Picture Vocabulary Test - Third Edition**. In*.* Circle Pines, MN: American Guidance Service, Inc; 1997.

23. Catts H: **Speech production/phonological deficits in reading disordered children**. *Journal of Learning Disabilities* 1986, **19**:504-508.

24. Lewis BA, Freebairn LA, Hansen AJ, Miscimarra L, Iyengar SK, Taylor HG: **Speech and language skills of parents of children with speech sound disorders**. *Am J Speech Lang Pathol* 2007, **16**(2):108-118.

25. Woodcock R: **Woodcock Reading Mastery Tests-Revised**. In*.* Circle Pines, MN: American Guidance Service; 1987.
