# Supplementary material for: Feature-driven classification reveals potential comorbid subtypes within childhood apraxia of speech
Source: BMC Pediatr. 2020 Nov 13;20:519. doi: 10.1186/s12887-020-02421-1 (PMC7664029; doi:10.1186/s12887-020-02421-1)
Supplement: Supplementary file 2 — Additional file 2. [file 12887_2020_2421_MOESM2_ESM.docx]

SUPPLMENTAL TABLES

**Supplemental Table 1: Descriptive Summary of CAS Individuals**

| Age at first assessment (mean and standard deviation) | | Sex (proportion female) | SES (proportion in each stratum) | | Language Impairment (proportion with trait) | Reading Difficulty (proportion with trait) |
| --- | --- | --- | --- | --- | --- | --- |
| Overall^1^  EOWPVT  GFTA  NWR  PPVT  WRMT-AT  WRMT-ID  DDK | 5.9 (2.5)  7.1 (2.3)  8.9 (5.1)  10.0 (2.8)  7.1 (2.3)  10.2 (2.7)  10.2 (2.7)  6.3 (2.3) | 0.19 | 1  2  3  4  5 | 0  0.03  0.42  0.32  0.23 | 0.94 | 0.58 |

^1^Mean age for first visit; n = 31

**Supplemental Table 2: Descriptive Summary of Typically developing children (controls)**

| Age at first assessment (mean and standard deviation) | | Sex (proportion female) | SES (proportion in each stratum) | | Language Impairment (proportion with trait) | Reading Difficulty (proportion with trait) |
| --- | --- | --- | --- | --- | --- | --- |
| Overall^1^  EOWPVT  GFTA  NWR  PPVT  WRMT-AT  WRMT-ID  DDK | 7.9 (3.6)  9.6 (3.2)  8.3 (3.6)  10.0 (2.0)  9.6 (3.2)  10.0 (2.0)  10.0 (2.0)  8.4 (3.0) | 0.63 | 1  2  3  4  5 | 0  0.14  0.14  0.71  0 | NA | 0.13 |

^1^Mean age for first visit; n = 8

**Supplemental Table 3. Comparison of male versus female children with CAS**

| **Clinical Traits** | **Females (n = 6)** | | | | **Males (n = 25)** | |  | |
| --- | --- | --- | --- | --- | --- | --- | --- | --- |
|  |  | |  |  |  |  |  |  |
|  | Proportion  exhibiting trait | | | | Proportion  exhibiting trait | | Fisher exact P-value |  |
| Problems with Feeding Eating | 0.33 | | | | 0.24 | | 0.63 |  |
| Little vocal play or babbling | 0 | | | | 0.16 | | 0.56 |  |
| Family history of communication disorders | 1 | | | | 0.96 | | 0.99 |  |
| Delayed language onset | 0.33 | | | | 0.76 | | 0.067 |  |
| Gross motor incoordination | 0 | | | | 0.36 | | 0.14 |  |
| Fine motor incoordination | 0.17 | | | | 0.32 | | 0.64 |  |
| Body dyspraxia body awareness in space | 0 | | | | 0.08 | | 0.99 |  |
| “Soft” Neurological signs | 0 | | | | 0.16 | | 0.56 |  |
| Sensory processing issues | 0.17 | | | | 0.12 | | 0.99 |  |
| Dysarthria | 0.17 | | | | 0.20 | | 0.99 |  |
| Limited repertoire of sounds | 0.5 | | | | 0.44 | | 0.99 |  |
| LI | 0.83 | | | | 0.96 | | 0.35 |  |
| Speech | 1 | | | | 1 | | 1 |  |
| Read | 0.50 | | | | 0.60 | | 0.68 |  |
| Spell | 0.33 | | | | 0.56 | | 0.39 |  |
| **Tests** | Mean (sd) [median] | | | | Mean (sd) [median] | | Mann-Whitney Exact P-value |  |
| NWR | -0.78 (0.8) [-0.57] | | | | -1.16 (0.8) [-1.43] | | 0.26 |  |
| DDK | | -3.9 (6.1) [-0.97] | | -8.8 (7.3) [-7.9] | | 0.07 | | |
| GFTA | -0.75 (1.6) [-1.26] | | | | -0.92 (0.7) [-1.08] | | 0.62 |  |
| PPVT | 0.06 (1.8) [0.17] | | | | -0.24 (1.1) [0] | | 0.62 |  |
| EOWPVT | -0.60 (1.3) [-1.13] | | | | -0.08 (1.1) [0] | | 0.32 |  |
| WRMT-ID | -0.74 (1.9) [-0.70] | | | | -0.93 (0.9) [-0.93] | | 0.74 |  |
| WRMT-AT | -0.32 (1.3) [-0.23] | | | | -0.98 (1.1) [-0.80] | | 0.28 |  |

**Supplemental Table 4. Descriptive statistics for last assessment of articulation, vocabulary, and reading tasks by cluster**

| Variable | Cluster 1 (High severity)  (n = 10) | Cluster 2 (Moderate severity)  (n = 11) | Cluster 3 (Low severity)  (n = 10) | Cluster 4 (Controls) (n = 8) | Kruskal-Wallis  P-value^1^ | Mann-Whitney exact  P-value^2^ | Mann-Whitney exact  P-value^3^ | Mann-Whitney exact  P-value^4^ | Mann-Whitney exact  P-value^5^ | Mann-Whitney exact  P-value^6^ |
| --- | --- | --- | --- | --- | --- | --- | --- | --- | --- | --- |
| EOWPVT | -0.93 (1.11)  [-0.87] | 0.16  (1.01) [0.27] | 0.05 (0.78)  [0.03] | 1.08  (0.64) [0.97] | 0.003 | 0.04 | 0.67 | 0.02 | 0.03 | 0.09 |
| GFTA | -0.49 (1.45)  [-0.57] | -0.53 (1.55)  [-1.27] | -0.71 (1.46)  [-1.27] | 1.68  (1.10) [2.33] | 0.02 | 0.99 | 0.98 | 0.006 | 0.69 | 0.02 |
| NWR | -1.82 (0.98)  [-2.11] | -2.04 (0.97)  [-2.09] | -0.60 (0.91)  [-0.91] | 0.01 (1.48) [0.52] | 0.005 | 0.76 | 0.004 | 0.27 | 0.04 | 0.007 |
| PPVT | -0.77 (0.96)  [-0.80] | 0.22  (0.58) [0.20] | 0.39 (0.79)  [0.17] | 0.81 (0.70) [0.80] | 0.004 | 0.009 | 0.93 | 0.21 | 0.01 | 0.09 |
| WRMT-AT | -1.30 (0.73)  [-1.00] | -1.04 (0.86)  [-1.27] | 0.07 (0.62)  [0.20] | 0.18 (0.76) [0.07] | <0.001 | 0.64 | 0.003 | 0.92 | <0.001 | 0.009 |
| WRMT-ID | -1.73 (0.76)  [-1.83] | -1.05 (0.61)  [-1.00] | -0.03 (0.70)  [0.10] | 0.09 (0.48) [0.17] | <0.001 | 0.05 | 0.007 | 0.85 | <0.001 | <0.001 |
| DDK | -2.40  (2.1)  [-1.65] | -1.10  (1.4)  [-1.27] | -0.83  (0.63)  [-0.67] | -0.40  (1.16)  [-0.23] | 0.07 | 0.28 | 0.60 | 0.32 | 0.02 | 0.31 |

Includes both CAS and controls. Values refer to the last available assessment and are shown as mean (sd) [median]. P-values shown are not adjusted for multiple testing. ^1^Comparison of distribution across all four clusters. ^2^Comparison of distribution medians between clusters 1 and 2. ^3^Comparison of distribution between clusters 2 and 3. ^4^Comparison of distribution between clusters 3 and 4. ^5^Comparison of distribution between clusters 1 and 3. ^6^Comparison of distribution between clusters 2 and 4.

**Supplemental Table 5: Age Distribution for Language and Reading Assessments by Cluster**

|  | Assessment | |
| --- | --- | --- |
| Cluster | Reading (WRMT-AT and WRMT-ID)  Mean (sd) | Language (PPVT and EOWPVT)  Mean (sd) |
| Controls | 10.0 (2.0) | 9.6 (3.2) |
| Mild | 10.3 (3.0) | 6.7 (2.8) |
| Moderate | 10.4 (3.6) | 7.5 (1.9) |
| Severe | 10.0 (1.2) | 7.0 (2.2) |

**Supplemental Table 6. Prevalence of ADHD by cluster group**

|  | **Severe (N=10)** | **Moderate (N=11)** | **Mild (N=10)** | **Control (no CAS) (N=8)** |
| --- | --- | --- | --- | --- |
| Number of children with reported ADHD (percentage) | 8 (80%) | 5 (45%) | 2 (20%) | 1 (12.5%) |

Fisher’s exactly p-value=0.013

**Supplemental Figure 1. Distribution of NWR z-scores in CAS children and unaffected controls**

**
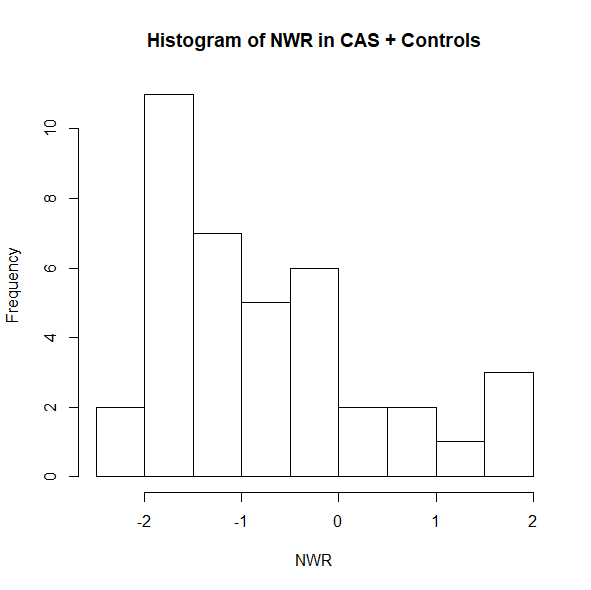
**

**Supplemental Figure 2. Illustration of cluster analysis with and without controls**

| **Clustering Dendrogram with Controls Included** | **Clustering Dendrogram with Controls Excluded** |
| --- | --- |
| **** | **** |

**Supplemental Figure 3. Distribution of PIQ scores by CAS comorbidity subtype cluster**
